# The Odor Context Facilitates the Perception of Low-Intensity Facial Expressions of Emotion

**DOI:** 10.1371/journal.pone.0138656

**Published:** 2015-09-21

**Authors:** Arnaud Leleu, Caroline Demily, Nicolas Franck, Karine Durand, Benoist Schaal, Jean-Yves Baudouin

**Affiliations:** 1 Équipe Éthologie développementale et psychologie cognitive, Centre des Sciences du Goût et de l’Alimentation, UMR 6265 CNRS–UMR 1324 INRA–Université Bourgogne-Franche-Comté, Dijon, France; 2 Centre de Dépistage et de Prises en Charge des Troubles Psychiatriques d’Origine Génétique, Centre Hospitalier le Vinatier, Bron, France; 3 Centre de Neuroscience Cognitive, UMR 5229 CNRS, Institut des Sciences Cognitives, Université Lyon 1, Lyon, France; 4 Service Universitaire de Réhabilitation, Centre Hospitalier le Vinatier, Bron, France; 5 Institut Universitaire de France, Paris, France; University of Montreal, CANADA

## Abstract

It has been established that the recognition of facial expressions integrates contextual information. In this study, we aimed to clarify the influence of contextual odors. The participants were asked to match a target face varying in expression intensity with non-ambiguous expressive faces. Intensity variations in the target faces were designed by morphing expressive faces with neutral faces. In addition, the influence of verbal information was assessed by providing half the participants with the emotion names. Odor cues were manipulated by placing participants in a pleasant (strawberry), aversive (butyric acid), or no-odor control context. The results showed two main effects of the odor context. First, the minimum amount of visual information required to perceive an expression was lowered when the odor context was emotionally congruent: happiness was correctly perceived at lower intensities in the faces displayed in the pleasant odor context, and the same phenomenon occurred for disgust and anger in the aversive odor context. Second, the odor context influenced the false perception of expressions that were not used in target faces, with distinct patterns according to the presence of emotion names. When emotion names were provided, the aversive odor context decreased intrusions for disgust ambiguous faces but increased them for anger. When the emotion names were not provided, this effect did not occur and the pleasant odor context elicited an overall increase in intrusions for negative expressions. We conclude that olfaction plays a role in the way facial expressions are perceived in interaction with other contextual influences such as verbal information.

## Introduction

The recognition of emotional states from facial expressions is not a purely visual process that consists of linking others’ facial expressions with previously encoded visual representations of discrete facial categories. The process also involves the integration of many types of contextual information that influence the way facial expressions are perceived (for reviews, see [1–3]). An illustrative example is the famous Lev Kuleshov effect, in which the exact same inexpressive face of an actor is differently interpreted when it is displayed after exposure to a scene representing a meal on a table, a child resting within a coffin, or a sexy woman lying on a sofa. The scientific literature abounds in observations on the recognition of facial expressions integrating various types of contextual information. For example, the perception of facial expression is influenced by the emotion conveyed by concurrent body posture [4,5], voice [6–9], surrounding visual scenes [10–12], social situations [13], and contextual sentences [14,15]. In some cultures, surrounding faces can also act as modulating factors [16].

The perception of emotion in faces also integrates several types of internal factors. For example, the way people perceive emotions in facial expressions depends on mimicry behaviors [17,18], social anxiety level [19], emotional state [17,20], social stereotypes [21], and the cultural environment [22–24]. Simply ascribing emotions to categories has been described as having a beneficial or shaping contextual effect ([25–28]; for a discussion, see [29]).

In that context, emotional cues carried by odors may be a potent factor in regulating the perception of facial expression. Chemosignals intervene in a large number of emotional responses, without the need for allocation of conscious attentional resources [30]. An increasing number of studies indicate that olfaction does not play a minor role because it modulates the processing of information by the “major” senses (e.g., [31,32]). Olfaction is strongly involved in multisensory social interactions (for reviews, see [33,34]), for example, by modulating or orienting the responses to faces (e.g., [35–38]). Such multisensory phenomena involving odor cues originate in early infancy [39]. With regard to emotions specifically, the olfactory system has close connections to the emotional brain (e.g., the amygdala, insula, and orbitofrontal cortex (OFC); [40,41]). These connections are not silent because olfaction modulates the cerebral response of several regions also known to process emotional information conveyed by faces, such as the insula [42], the amygdala ([43–45]; for a review, see [46]), and the OFC [43,45,47].

At a behavioral-cognitive level, the effect of chemosignals in the processing of facial expressions has been repeatedly demonstrated with body odors (e.g., [48–52]). Overall, these studies have shown that anxiety- or fear-related chemosignals favor the perception of related expressions and interfere with the perception of opposite expressions, most notably in the case of ambiguous expressions. A few studies have further reported that the context created by arbitrary surrounding odorants influences the perception of facial expressions ([53–55]; see also [38]). For example, Leppänen and Hietanen identified the faster and more accurate recognition of happy faces in pleasant odor contexts (lemon, strawberry, or vanilla) than in aversive (pyridine) odor or no-odor contexts [53]. Disgust was not influenced by the odor context in this study. Conversely, Seubert and collaborators identified slower and less accurate recognition of smiling faces with odor priming, regardless of the valence of the odor (vanillin vs. hydrogen sulfide) [54]. They also reported an enhanced speed and accuracy for disgust face recognition in their two studies, regardless of the hedonic valence of the odor context [54,55]. These responses were associated with the modulation of several brain regions–including the insula–in the odor context [55].

Thus, the few studies using arbitrary odorants do not provide a clear picture of the way non-body odors may influence the perception of facial expressions. More specifically, it is unclear whether odor-based modulations occur only for emotionally congruent olfactory-visual stimuli or whether the effect is more “global”, and affects several expressions (see the diverging reports by [53] and [54,55]). The precise cognitive processes that underlie these effects are unknown and require further investigation.

In the present study, we aimed to explore the ways in which the context created by non-body odors intervenes in the processing of facial expressions. More specifically, we intended to assess whether and how the odor context can influence the matching of an observed facial pattern with the corresponding visual representation. Several predictions can be made here. First, the odor context might favor the extraction of facial information through priming-like or fluency-like effects [53–55]. Accordingly, olfactory stimuli could pre-activate visual representations of emotional expressions through intersensory integration processes. Any further or concomitant perception of the corresponding facial expression would then be facilitated. To this end, we examined whether the activation of expression representation would need less visual information (i.e., decrease in the intensity of expression needed to accurately perceive the emotion). We also tested (i) whether the effect of odorants was specific to facial expressions that share the same emotional meaning (i.e., aversive odorant-disgust face and pleasant odorant-smiling face) or extended to (some) other expressions (anger, fear, and sadness), and (ii) whether the effect of the odor context resulted from a congruency effect (facilitation of related odor-expression processing), an incongruence effect (interference for any or some unrelated expressions), or both. These two points have received contradicting answers in previous studies [53–55].

The second prediction is that the odor context might act as clarifying information [53,56]. Different emotions are expressed by common facial features, sometime acting in the same way. For example, the action of brow lowering is performed during anger, fear and sadness [57], but it is also frequently used when people simulate disgust [58]. Thus, multiple facial actions have to be considered to accurately identify the resulting facial expression and the emotion associated with it. By inducing the processing of a specific expression, the odor context may orient the cognitive system toward the relevant facial cues, but it could also inhibit the processing of irrelevant cues. This phenomenon would reduce the perceptual load of processing irrelevant information and limit the false perception of another expression. To illustrate this point, disgust, anger, and sadness are expressions that are frequently confused (e.g., [59]). One way to improve their recognition is to disentangle them by rapidly processing both common and distinctive facial actions. The odor context might promote such a process, for example, by biasing the cognitive system toward facial cues underlying the typical disgust pattern (e.g., wrinkled nose + lowered eyebrows + raised cheek + compressed lids + raised upper lip) in an aversive odor context. The same might occur for cues of happiness in a pleasant odor context. Conversely, for emotionally incongruent expressions, the odor context may reduce facial expression identification because the cognitive system is oriented toward cues that are irrelevant for these expressions; for example, in an angry face, some “disgust” cues (e.g., lowered eyebrows, compressed lids) might receive more attention in an aversive odor context.

Finally, although top-down mechanisms clearly influence the processing of olfactory or visual inputs, little is known about the way such processes intervene in the *integration* of olfactory and visual inputs. Certainly the processes operating in the interactions between visual and olfactory cues are shaped by information from language (emotion names), which helps to categorize facial expression by way of language-induced categories [25,28,29]. Similarly, the olfactory context might also help shape the emotional space and may then compete with the top-down influence of language. The significance of the odor context may then be reduced by providing the names of the emotion categories. The odor environment might also act different depending upon whether the facial categories are pre-established by emotion names or must be defined on the sole basis of visual information. In the first case, olfactory information may work according to language-induced categories. In the second case, olfactory information may participate in the shaping of the emotional space, for example by inducing a classification in terms of olfaction-related emotional categories (e.g., pleasant vs. unpleasant).

To tackle these issues, we studied whether the odor context influences the visual matching of ambiguous low-intensity facial expressions with non-ambiguous expressions displayed by the same person. The participants were presented with (i) a target face whose ambiguity could vary according to different expression intensities and (ii) non-ambiguous original, neutral and expressive faces (anger, disgust, fear, sadness, or happiness) simultaneously. They were required to identify which non-ambiguous face the target face looked more like. The odor context was varied to be pleasant (strawberry), aversive (butyric acid), or a no-odor control context. We hypothesized that the odor context would influence the perception of expression in low-intensity expressive faces in two ways. First, in the case of congruency between odorant and expression (i.e., aversive-disgust and pleasant-happiness), we hypothesized that the odor context would decrease the amount of facial information needed to recognize the expression. Consequently, the ambiguous and non-ambiguous expressions should be correctly matched from a lower intensity of expression in the target face. An incongruent effect was also envisaged, with a higher intensity of expression needed to recognize expressions that are not congruent with the odorant. Second, we hypothesized that the odor context may decrease the number of false perceptions (or intrusions) arising from seeing another expression than the one actually used for the target faces (e.g., perceiving sadness in an ambiguous neutral-disgust face). To evaluate whether providing information about emotion names/categories influenced the way olfaction is integrated, half of the participants were explicitly told the emotion names of the non-ambiguous expressions, whereas the other half were not and performed the task on the basis of visual information only.

## Methods

### Participants

**Fig 1:**
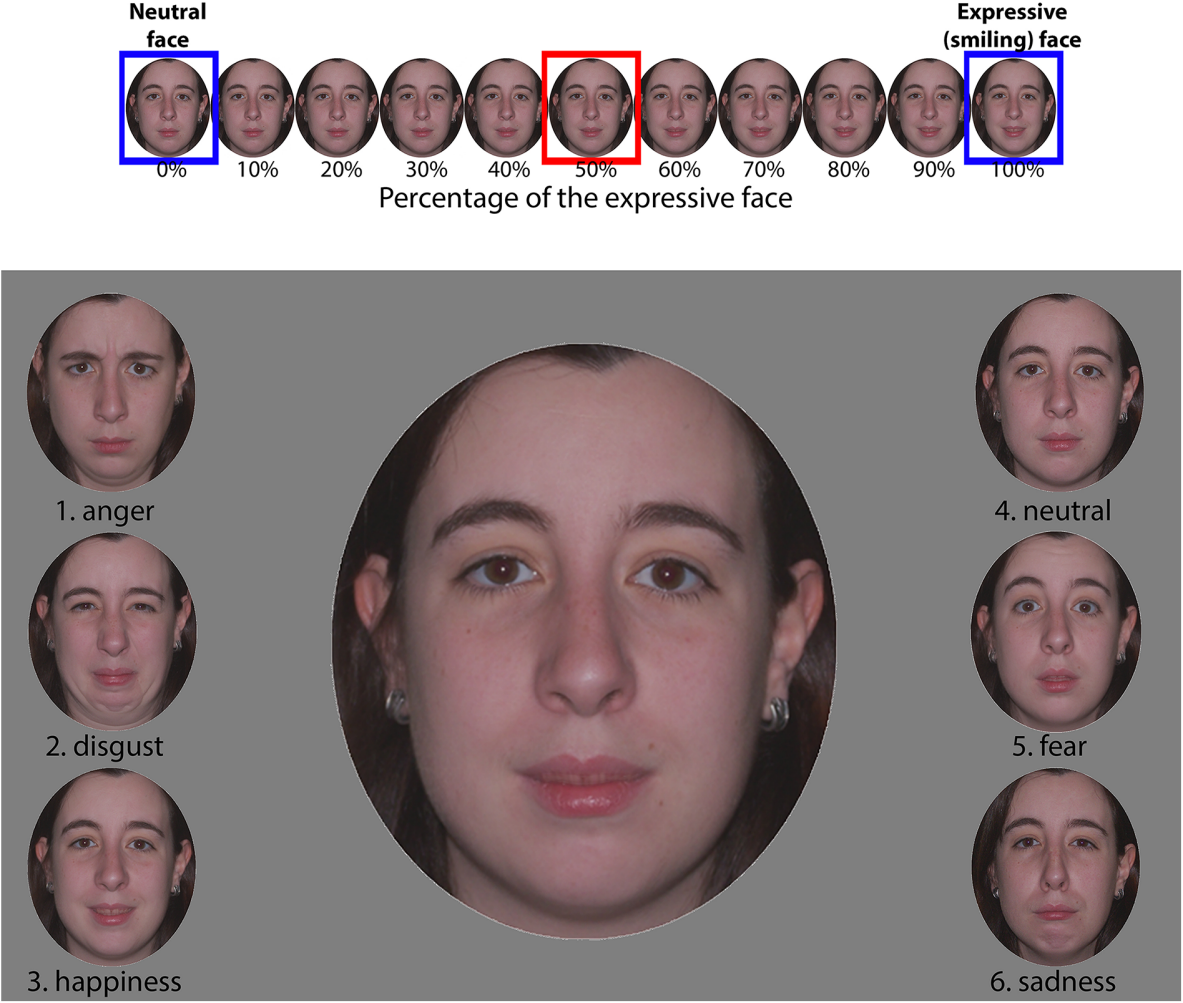
Face stimuli and procedure. Illustration of the morph continua (top) and of the display of ambiguous and non-ambiguous expressions during a single trial in the main experiment (bottom). Top: the expressive faces (smiling in the illustration) were morphed with the neutral faces in steps of 10%. Bottom: during a trial, the central large face was one of the morph levels (50% of the smiling face in the illustration; see the red rectangle in the continuum). The lateral smaller faces represent the extrema of all continua for the model (e.g., the “4. neutral” and “3. happiness” faces at the bottom were the faces in blue rectangles in the continuum). The participants were instructed to determine which lateral face the central one more resembled. For half the participants, the emotion names were removed.

Forty-eight participants took part in the experiment (31 females; mean age: 21.8 years, range: 18–37 years). All participants had normal or corrected-to-normal visual acuity, and none reported any prior neurological or psychiatric disorders. All participants provided written informed consent prior to beginning the experiment and were compensated for their participation. This study was approved by the CPP (Comité de Protection des Personnes) Lyon Sud-Est IV and conducted in accordance with the Declaration of Helsinki. The individual displayed in Fig 1 has given written informed consent for her photograph to be published.

### Stimuli

#### Face stimuli

The face material was adapted from previous studies, where it was pretested among eleven participants and chosen because at least ten subjects agreed on the emotion expressed (e.g., [60,61]). The expression set consisted of 24 color pictures of 4 models (2 females), each picture corresponding to one emotional expression among 6 (anger, disgust, happiness, fear, sadness and neutrality). Information about the background and body was removed by cropping the face into a medallion shape, and each stimulus was displayed on a mid-level grey background. For each emotional expression, an increase in intensity was designed using a linear continuum of morphs created by combining the expression with neutrality. Ten pictures were extracted for each continuum (every 10%, from 10 to 100% of the original expression). After this procedure, we obtained 51 pictures for each of the 4 models (10 morph levels × 5 emotions + the neutral face). In the first step, a pilot study was conducted with 16 control participants (11 females; mean age: 24.4 years, range: 19–31 years). The design was similar to the present experiment (see below Setting and Procedure section), but no odorants were used. The goal of the pilot study was to create a new set of stimuli by equating the perception threshold for the different models and emotions around an intermediate intensity of expression. Hence, from the pilot study data, we computed the lowest expressive face intensity that corresponded to the perception threshold in each continuum (for details on computation of threshold, see the Data analyses section below). The corresponding morph level was multiplied by two, and a new continuum was designed from this two-time-threshold morph level. Thus, we used the two-time-threshold morph levels as “original” expressive faces and created new continua (with steps of 10%) where these levels were used to represent “100% expression” and morphed with the neutral face. For example, if the participants in the pilot study detected happiness at the 30% morph level for a model, we considered the picture corresponding to the 60% morph level to represent the 100% morph level in the present study and generated a new continuum centered on the lowest intensity needed to perceive the expression. The final set of stimuli was thus composed of 51 pictures for each of the 4 models (10 morph levels × 5 emotions + the neutral face). An example of the continua used in the experiment is illustrated in Fig 1 (top part).

#### Odor stimuli

Two odor stimuli were selected for their contrasted *a priori* hedonic valence. A fruity odor, strawberry (alimentary quality, Meilleur du Chef, Maignon, France), and the odor of butyric acid (Sigma-Aldrich, Saint-Quentin-Fallaviers, France), conveying a cheesy quality. Both of these odorants have often been used to elicit positive or negative affective states in previous studies of emotion [62–64]. Although the strawberry odor was used undiluted, butyric acid was diluted in scentless mineral oil to reach a concentration of 10^−5^ v/v. Twenty-four hours before each experiment, the two odorants were absorbed into the polyurethane foam-cover of the microphones of two distinct sets of headphones. Doses of 50 and 100 μL (one and two drops) were used for strawberry and for butyric acid, respectively. The subjective intensity equivalence of both odorants was equated and assessed by four adult raters. The no-odor control stimulus consisted of the same headphone-microphone set but without odorant.

### Setting and Procedure

The participants sat approximately 60 cm from a computer screen with headphones affixed to their heads. In each trial, they were told to categorize the expression of the large target face (15 × 12 cm) displayed at the center of the screen by selecting one of 6 smaller faces (5 × 4 cm) displayed on the left (3 small faces: anger, disgust, and happiness) and the right sides (3 small faces: neutral, fear, and sad) of the screen (see the bottom part of Fig 1 for an illustration). The central target face was one picture among the 51 comprising the set of stimuli for one model. Accordingly, its emotional expression could be more or less intense. The 6 small faces were the neutral and the most expressive faces (100% morph level) used to build the continua from which the central face was extracted. For half of the participants, a digit was written under the small faces. These participants were not told about the emotion categories of the faces before or during the experiment. For the other half, the same digits accompanied the small faces, but the names of emotion categories were also written after the digit to make the categories explicit. The participants were told to respond to each screen display orally in a loud voice into the microphone in front of their mouth (and directly under their nose) by using the digit for the first half of participants and the name for the second half. As the only purpose of the microphone was to deliver the odorants, the responses were recorded by the experimenter, who pressed the corresponding key on the numerical pad of the keyboard. Stimuli were displayed until the experimenter pressed the key, and it was argued that the keyboard was used to tag the oral responses in the audio recording sequence.

Each participant performed 7 blocks of trials, the first block being for training purposes. In each block, the 51 stimuli from a model were displayed randomly, and a single participant was exposed to a single model through all the blocks (different participants performed the task with the 4 models). Between each block, the participants took breaks during which they were instructed to perform the Bells test: they were required to cross out as many bells as they could in one minute on a sheet of paper where many figures were illustrated [65]. The purpose was to make them remove the headphones and to draw their attention away from the experimental design and the experimenter. During this time, the experimenter replaced the headphones with another set. Thus, the odorant within the microphone was varied across blocks, with the three odor contexts rotated between the three first blocks (the same order being repeated during the three final blocks). All possible orders were performed and counterbalanced between participants.

At the end of the experiment, the participants were interviewed regarding whether they noticed something special during the experiment, then more precisely regarding their degree of awareness of odors in the environment. One participant spontaneously reported having smelled an odor, while eighteen other participants only reported smelling an odor when they were specifically asked for. The twenty-nine remaining participants did not report smelling any odors. None of the participants noticed the exact source of the odorants (the microphone), but reported that it was somehow linked to the room or the experimenter. Importantly, no participants established that the presence of odors was related to experiment purposes. After this interview, they were asked to smell the foam-cover of the three microphones to evaluate whether they were able to detect an odor, and if so, to identify the odors. All participants were able to detect both odors, sixteen correctly identified strawberry and four correctly described butyric acid as a cheesy odor. Finally, they were questioned about the hedonic valence of each odor by inquiring whether it was pleasant, neutral, or unpleasant. All included participants judged butyric acid as an unpleasant odor and strawberry as a pleasant odor. Three additional participants were initially included in the study but were discarded from the analyses because one of them judged strawberry as an unpleasant odor, another one judged strawberry as a neutral odor, and the last judged butyric acid as a neutral odor.

### Data analyses

The first step in the analyses consisted of computing the response rates for each possible response in each morph level from each continuum in each block. A preliminary analysis was performed on the response rates for the most intense expressive faces (100% morph level) and neutrality to ensure that participants were able to accurately categorize the emotions in the non-ambiguous stimuli. The second step consisted of computing three indices derived from the response rates: intensity of expression for correct perception, percentage of intrusions, and percentage of times a given expression intruded.

The *intensity of expression for correct perception* corresponded to the lowest morph level within a continuum for which participants correctly categorized the expression. It was corrected in case of false response for higher intensities of expression by averaging this morph level with the next level for which the participants responded correctly. For example, if the lowest intensity for which a participant responded “happiness” was for the 30% morph level within the happiness continuum (with no errors for higher levels), the intensity of expression for correct perception was 30%. However, if the participant responded correctly “happiness” for the 30% morph level, but made an error for the 40% morph level and not for the 50% morph level, the intensity for correct perception was (30% + 50%)/2 = 40%. The *percentage of intrusions* was the percentage of responses that did not correspond to an expression used within the continuum (for example, the sum of the percentage of responses “anger”, “disgust”, “fear”, and “sad” for faces extracted from the neutral-happy continua). We also analyzed the *percentage of times a given expression intruded* into continua in which it was not used. For example, the percentage of times happiness intruded into the continua generated from the 4 other expressions. All indices were first computed for each block separately, and then averaged for the two blocks of the same odor condition.

Three-way analyses of variance were performed on each of the 3 indices with Group (emotion names vs. without emotion names) as a between-subjects factor, and Expression (anger vs. disgust vs. fear vs. sadness vs. happiness) and Odor context (no-odor vs. pleasant vs. aversive) as within-subjects factors.

## Results

As expected, preliminary analyses showed that the participants were very accurate (i.e., close to ceiling) in the correct perception of both neutral faces (95.5%) and the most intense expressive faces (from 95.8% of correct perception for sadness to 99.7% for happiness, with 97.2%, 99.0%, and 99.3% for disgust, anger, and fear, respectively). The odor contexts or groups did not significantly modulate accuracy here.

### Intensity of expression for correct perception

In accordance with our prediction that the intensity of expression needed to correctly perceive emotions would be diminished by providing corresponding names, participants provided with emotion names perceived the expressions at a lower percentage than those who were not (41.9% vs. 45.9%). However, the main effect of the Group factor was not significant [F(1,46) = 2.77]. Despite our attempts to standardize the data across expressions, the main effect of the Expression factor was significant [F(4,184) = 8.19, p < .0001, η_p_^2^ = .15] and showed that the percentage for happiness (38.6%) was lower than the percentages for the other expressions [from 43.9% to 47.3%; F(1,46) = 21.53, p < .0001]. The other expressions did not differ [F(3,138) = 1.97].

**Fig 2:**
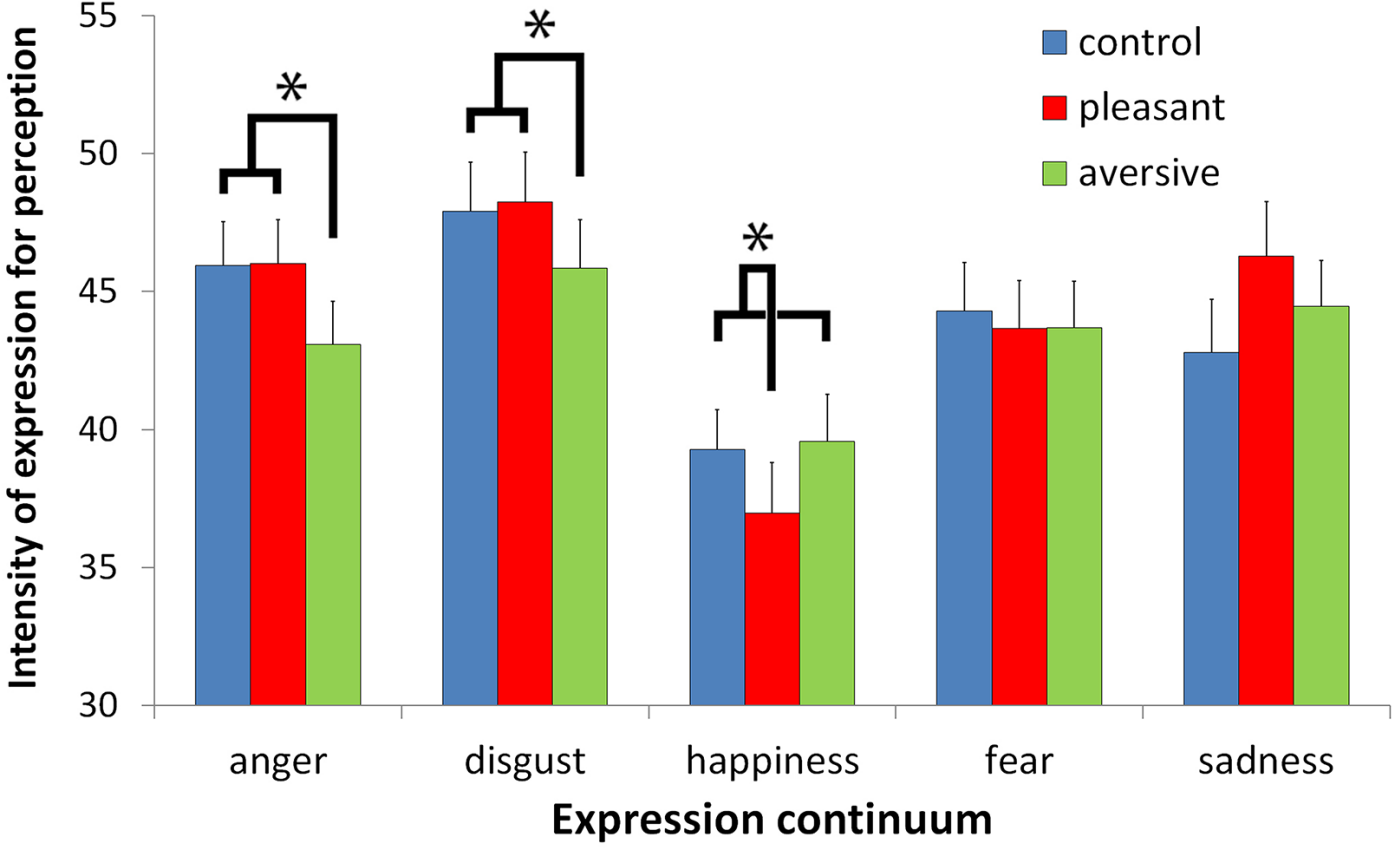
Intensity of expression for correct perception. Mean minimum intensity of expression (in percentage of expression) in the morphed target faces for correct perception of the expression, according to Odor context and Expression continua (error bars are standard errors of the means).

Interestingly, for our other prediction that the odor context would decrease the intensity of expression for correct perception in case of congruence between the odor and the emotion and, possibly, increase it in case of incongruence, the main effect of the Odor context was not statistically significant [F(2,92) = 1.11], but the interaction between Expression and the Odor context was [F(8,368) = 3.11, p < .01, η_p_^2^ = .06; see Fig 2]. The odor context significantly modulated the intensity needed for the perception of anger [F(2,92) = 3.76, p < .05], disgust [F(2,92) = 3.31, p < .05], and happiness [F(2,92) = 3.59, p < .05] but not for fear and sadness [F(2,92) = .19 and F(2,92) = 2.35, respectively]. For anger and disgust, the percentage of expression for correct perception was lower in the aversive odor context (43.1% for anger and 45.8% for disgust) than in both the control and pleasant odor contexts [for anger: 45.9% and 46.0%, respectively, F(1,46) = 10.34, p < .01; for disgust: 47.9% and 48.2%, respectively, F(1,46) = 6.27, p < .05]. Conversely, for happiness, the percentage of expression was lower in the pleasant odor context (37.0%) than in both the control and aversive odor contexts [39.3% and 39.6%, respectively, F(1,46) = 7.03, p < .05]. Nevertheless, no incongruent effects were noted: the differences between the control and incongruent odor contexts were not significant for the three expressions (all Fs<1). Fig 2 indicates that the percentage for correct perception of sadness also increased in the pleasant odor context, but neither the main effect of an odor context (as already stated above) nor the direct comparison of control and pleasant odor context reached statistical significance [F(1,46) = 3.85, p = .0559]. Exploratory descriptive analyses also suggested that the odor context effect for disgust tended to be larger for participants who were provided with the emotion names. By contrast, the odor context effect for anger tended to be larger for participants who were not provided with the names (S1 Fig). However, these descriptive effects were not confirmed by inferential analyses, as the interaction between Group, Expression and Odor context factors was not statistically significant (F<1).

### Percentage of intrusions

**Fig 3:**
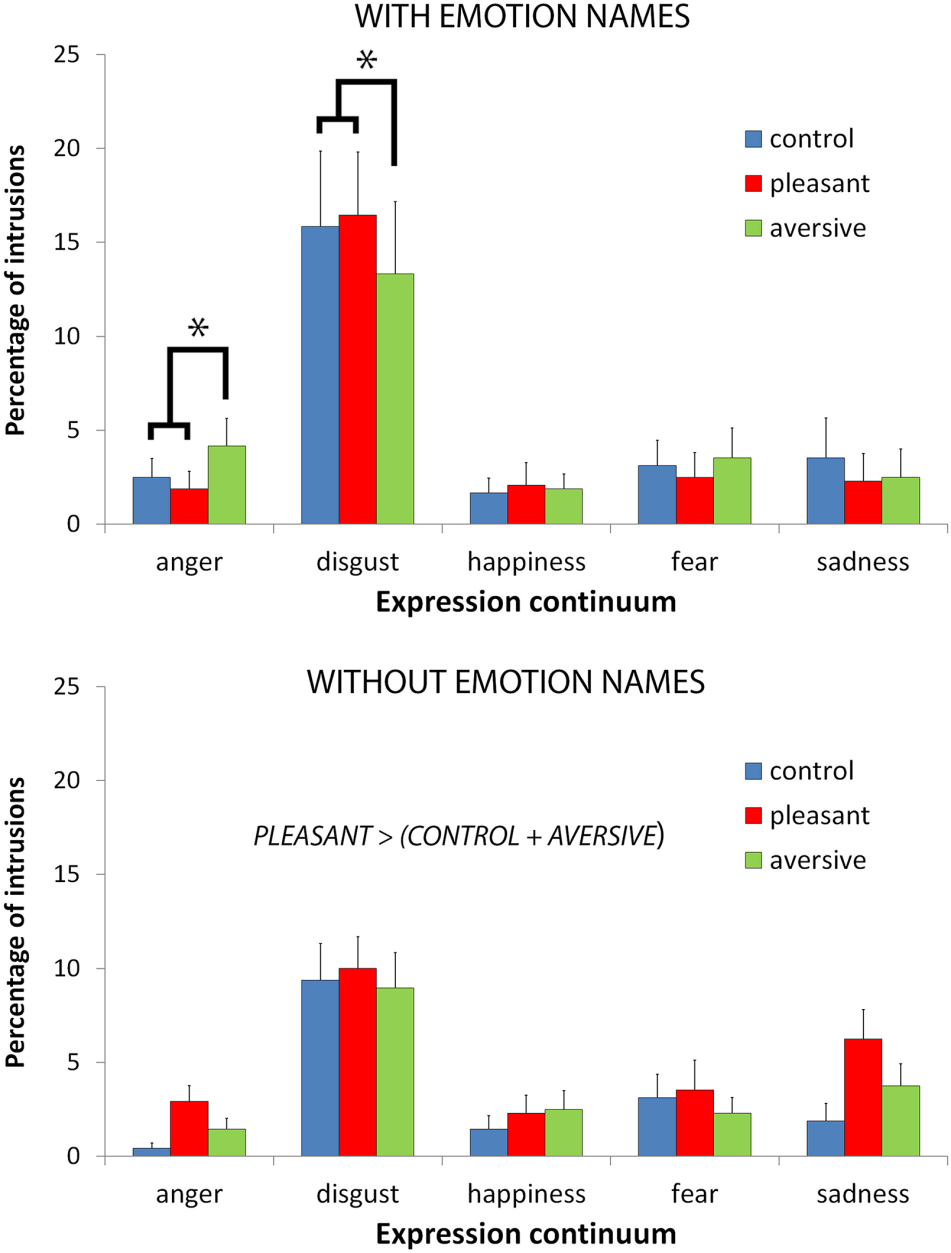
Percentage of intrusions. Mean percentages of intrusions of other expressions according to Odor context, Expression continuum, and Group (error bars are standard errors of the means).

We predicted that intrusions (i.e., the false perception of emotional expressions that were not used to make the low-intensity face) would be modulated by the influence of emotion names. Although the percentage of intrusions was not globally modulated by the presence of the emotion names (main effect of Group: F<1), more intrusions were observed in the disgust continuum, and this effect was more pronounced after providing emotion names (see Fig 3). Indeed, the main effect of the Expression factor was significant [F(4,184) = 26.08, p < .0001, η_p_^2^ = .36], indicating that the percentage of intrusions was larger for the disgust continuum (12.3%) than for other expressive continua [2.2% to 3.4%; F(1,46) = 34.45, p < .0001], which did not differ [F(3,138) = 1.72]. This effect was significant for both groups but was larger for the group provided with the emotion names [Expression x Group interaction: F(4,184) = 2.54, p < .05, η_p_^2^ = .05; effect of Expression for the group provided with the emotion names: F(4,184) = 21.76, p < .0001; for the group not provided with the emotion names: F(4,184) = 6.86, p < .0001]. Sadness was the most intruding expression in both groups on the disgust continuum (5% when the emotion names were not provided; 10.5% when they were provided) compared with the other expressions (happiness: 2.2% and 3.8%; anger: 1.6% and 0.6%; fear: 0.6% and 0.4%, respectively). See the analysis of intruding expressions in the next section for further details.

Importantly, we also predicted that intrusions would be modulated by the odor context and that distinct patterns of odor context effects might emerge when emotion names were provided or when they were not. Providing the names of emotional expressions significantly modulated the effect of the odor context: the main effect of the Odor context was not significant [F(2,92) = 2.11], but it was qualified by the Group factor [F(2,92) = 3.75, p < .05, η_p_^2^ = .08] and by the interaction of the Group and Expression factors [F(8,368) = 2.28, p < .05, η_p_^2^ = .05; see Fig 3]. Further analyses indicated that the odor context had distinct effects when the emotion names were provided but not when they were absent (in which case the odor context had a global effect). Indeed, the main effect of the Odor context was significant for the group with no emotion names [F(2,92) = 5.68, p < .01] but not for the other group [F<1]. The percentage of intrusions was higher in the pleasant odor context (5.0%) in comparison with the two other contexts (3.3% for the control context and 3.8% for the aversive odor context; F(1,46) = 7.74, p < .01), which did not differ (F(1,46) = 1.55). Conversely, the interaction between the Odor context and Expression was significant for the group with the emotion names [F(8,368) = 2.27, p < .05] but not for the other group [F(8,368) = 1.60]. The aversive odor context reduced the rate of intrusions in the disgust continuum by comparison to both the control and pleasant odor contexts [13.3% vs. 15.8% and 16.5%, respectively; F(1,46) = 4.76, p < .05]. At the same time, the aversive odor context enhanced the intrusion rate within the anger continuum by comparison to both the control and pleasant odor contexts [4.2% vs. 2.5% and 1.9%, respectively; F(1,46) = 7.38, p < .01]. For expressions that intruded less in the disgust continuum, the effect was mainly driven by sadness (mean difference between the aversive odor context and the two other contexts: -1%), happiness (-0.9%) and anger (-0.8%). For expressions that intruded more in the anger continuum, the effect was also mainly driven by sadness (+0.8%) and, to a lesser extent, by disgust (+0.5%), fear (+0.4%) and happiness (+0.2%). See the analysis of intruding expressions below for further details.

Interestingly, the pleasant odor context increased the rate of intrusions, whatever the emotional continuum, in the absence of emotion names (i.e., even for happiness). This conclusion is based on the non-significant interaction between the Odor context and Expression factors. An alternative explanation is possible. In the absence of category names, the participants may not have clearly partitioned the emotional space into discrete conceptually driven categories, as we hypothesized in the Introduction. More specifically, negative emotions could have been less sharply defined and the perception (and thus the pattern of intrusions) was more strongly shaped along a positive-negative dimension than when discrete conceptually defined negative expressions were clearly stated. By considering negative facial expressions as distinct instances, we may have missed this aspect in the previous analyses. Thus, we computed the interaction between the Odor context, Expression, and Group factors with two categories of facial expression: positive expression (i.e., happiness) and negative expressions (i.e., anger + disgust + fear + sadness). The interaction between the three factors was significant [F(2,92) = 3.50, p < .05, η_p_^2^ = .06]. Further decomposition of this interaction indicated that, contrary to the previous analysis with four negative expression categories, the interaction between Odor context and Expression was only significant for the group with no emotion names [F(2,92) = 4.51, p < .05; for the other group: F<1]. When the emotion names were not provided, the percentage of intrusions was higher for negative expressions in the pleasant odor context by comparison to both the control and aversive odor contexts [5.7% vs. 3.7% and 4.1%, respectively; F(1,46) = 8.43, p < .01]. The odor context had no significant effect on the percentage of intrusions for the positive expression of happiness (F<1).

### Percentage of times a given expression intruded

In the following analyses, we characterized which facial expression(s) more frequently intruded without considering the continuum in which it intruded, and evaluated whether the emotion names or the odor context modulated the nature of intruding expressions. The main effect of Expression was significant [F(4,184) = 7.57, p < .0001, η_p_^2^ = .14], with more frequent intrusions of sadness (2.6%) than of any other expression [from 0.5% to 1.2%, F(1,46) = 10.37, p < .01]. This effect was significant when participants were explicitly given the names of emotion categories but was not significant when they were not [Group x Expression interaction: F(4,184) = 2.70, p < .05, η_p_^2^ = .06; main effect of Expression for the group with the emotion names: F(4,184) = 9.31, p < .0001; for the group without the emotion names: F<1; see S2 Fig]. Sadness intruded most when the emotion names were provided in the disgust continuum (10.5%) than in the other continua (anger: 1.7%; happiness: 1%; fear: 1.3%). Taking into account the previous analysis of intrusions, this result indicates that the high level of intrusions for the disgust continuum, especially in the group with the emotion names, was mainly driven by intrusions of sadness.

The odor context did not significantly modulate the type of expression that intruded [Expression x Odor context interaction: F(8,368) = 1.53; Expression x Odor context x Group interaction: F(8,368) = 1.83]. Thus, the odor context appeared to neither reduce nor increase the intrusion of one expression into the others. As in the analysis of percentage of intrusions in the different expression continua, the main effect of the Odor context was not significant [F(2,92) = 2.11], but the interaction between the Odor context and Group was [F(2,92) = 3.75, p<05, η_p_^2^ = .08]. As this interaction effect mirrored the same interaction for the percentage of intrusions (i.e., significant effect of the odor context only for the group with no emotion names, with more intruding expressions in the pleasant odor context; see the previous analyses on the percentage of intrusions), we will not detail it further. The absence of interaction among the Odor context, Intruding expression and Group suggests that the increase in intrusions in the pleasant odor context for the group with no emotion names was not associated with a specific intruding expression (a descriptive effect was mainly observed for anger, disgust and happiness; see S2 Fig). Likewise, while we found that the odor context selectively modulated the rate of intrusions for angry and disgust faces when the emotion names were provided (see *Percentage of intrusions*), there was no statistical evidence that it selectively modulated the intrusion of specific expression(s) (e.g., sadness, disgust or happiness).

## Discussion

A major finding of this study is that the odor context significantly facilitated the perception of ambiguous low-intensity expressions of faces, especially when the emotional meaning of the odorant was congruent with the emotional meaning of the facial expression. Specifically, happiness was perceived at a lower intensity of facial expression when the participants were in the pleasant odor context. Conversely, disgust–but also anger–was perceived at a lower intensity of expression in the aversive odor context. Thus, the perception of facial expressions is not a purely visual process but also integrates cues from other sensory modalities, including olfaction. The emotional information conveyed by olfaction helped the participants to distinguish facial expressions when the cues exhibited a low level of expressivity. This observation supports the idea that intersensory integration appears to be driven by olfaction-facial expression emotional congruency [53], rather than by the global effect of the odor context [54,55]. This result also extends the congruency effect already reported for happiness by Leppänen and Hietanen [53] to disgust. However, it also suggests that an aversive odor context is “congruent” with the expression of anger. We will return to this point below.

Another noteworthy result of this study is the finding that providing emotion names had two top-down consequences. First, it tended to decrease the intensity of expression needed to recognize any emotion in faces. This suggests that providing emotion names is beneficial for accurate categorization of emotions in low-intensity expressive faces, but this may be because redundant information from visual and verbal cues leads to a stronger bias toward relevant facial configurations. The presence of verbal information may act as a shaping context that orients toward sharper and more restricted emotional categories (e.g., [29] for a discussion). Second, while the continuum for disgust was the continuum suffering the most intrusions in general, its rate of intrusions was amplified by the provision of emotion names and this effect was mainly driven by intrusions of sadness. The preliminary analyses indicated that disgust was well perceived in the most intense expressive faces (i.e., 100% morph level), even better than for sadness. Moreover, the stimuli were pretested and chosen because participants were able to accurately recognize the emotions. Thus, this high level of intrusions cannot be explained by poorly designed research material for the expression of disgust. A more likely explanation is that disgust is not often perceived with a low intensity in daily-life, as people are either disgusted or not disgusted with no intermediate levels. Therefore, artificially designed low-intensity disgust may not look like a natural expression of disgust, and may be perceived as another emotion, such as sadness. The greater intrusion of sadness when the emotion names were provided reinforces our previous interpretation that verbal information may act as a shaping context. Accordingly, the verbal cues may have driven the cognitive system to specifically search for a restricted set of emotions, thus increasing the opportunity to perceive sadness in ambiguous low-intensity disgusted faces.

Our consideration of intrusions has contributed to a better understanding of the way olfaction modulates the perception of facial expressions of emotion and whether it depends on the presence of verbal information. Indeed, distinct patterns of modulation by odors occurred whether the participants were provided with the emotion names or not. When the emotion names were provided, the intrusions on the disgust continuum were reduced by the aversive odor context, and intrusions were more frequent on the anger continuum. When the emotion names were not provided, no such effect occurred, and the odor context resulted in an overall increase of intrusions for negative expressions in the pleasant odor context. In other words, the odor context helped participants recognize low-intensity disgust when expression categories were shaped by words (though it also increased the ambiguity of anger). When the expression categories were not shaped by words, the pleasant odor context made distinguishing between negative expressions more difficult.

Another important conclusion of this study is that the intersensory influences reported here were only facilitation effects; as long as we considered the intensity of expression for the correct perception, we did not observed interference effects. An effect of incongruence between the odor context and expression factors was only reported for intrusions, and it was limited to two situations: for anger when the emotion names were provided (with more intrusions in the aversive odor context) and for all negative expressions when emotion names were not provided (with more intrusions in the pleasant odor context). Accordingly, incongruent effects for happiness reported by Leppänen and Hietanen [53] were observed by participants’ reaction times, and only congruent effects were reported with accuracy. However, they did not observe congruency effects for disgust despite a slight descriptive trend in accuracy. Thus, congruency effects for disgust in the present study may result from both the reduced intrusion of other expressions and a decreased intensity threshold of expression for correct perception.

Having demonstrated that emotionally contrasted odors modulate the processing of emotions in faces, the exact ways along which olfaction intervenes in the perception of facial expressions remains to be clarified. With regard of the current literature, two non-exclusive explanations can be proposed. ***First***, the odor context could pre-activate visual representations of facial expression through multisensory cognitive processes in multisensory and/or emotional brain regions [38,55]. This pre-activation may improve the ability of these processes to catch subtle emotional cues in ambiguous low-intensity facial expressions. Several brain structures may play such a role. For example, the amygdala and the OFC are known to respond to visual as well as to non-visual emotional stimuli [38,43–47]. The insula has also been reported to be activated by viewing or smelling disgusting stimuli, as this visceral sensorimotor structure is involved in both the feeling and the perception of disgust ([42]; see also [55]). In a similar fashion, the odor context may have involved or simulated emotional reactions through embodiment in somatosensory areas, allowing the matching between these emotional states and those perceived in the expressive faces (e.g., [66]). The variety of brain structures and their sensitivity to distinct emotional contents (e.g., the particular sensitivity of the insula to aversive/disgusting stimuli [42]) permit the suggestion that the location of the odor effects in the brain varies according to the emotional meaning of the odorant. It was also evident that several types of contextual information shape the way expression categories are processed by these structures [1,3]. In the present study, the influence of olfaction could have been mediated in different ways according to the context in which it intervened, working either through conceptual knowledge when the emotion names were present (i.e., enhancement of the sensibility/ability to perceive odor-related expression categories and modulation of confusion within these categories) or through olfaction-specific knowledge when the emotion names were absent (enhancement of the sensibility/ability to perceive odor-related expression categories but also overall enhancement of intrusions for other non-congruent categories).

A ***second*** explanation could be that the odor context involves the motor system by the way of mirror neurons (e.g., [67]). One could suggest that the odor context provoked facial micro-reactions in the participants [68]. Such micro-reactions may act as mimicking-like responses and facilitate the recognition of emotional facial expressions ([17,20]; for a review, see [69]). For example, blocking mimicry by asking the participant to bite a pen (which results in EMG activity across muscles at the level of the mouth) modulates the recognition of happiness, an expression that strongly relies on these same muscles ([18]; see also [70]). By inducing emotion-related micro-mimics, the odor contexts may have pre-activated the motor/mirror system, which may operate as priming processes on later facial expression perception. For example, the aversive odorant may have elicited the activation of nose wrinkling or brow lowering action units, whereas the pleasant odorant may have elicited the activation of smiling-related action units (e.g., lip corner puller, lips part, cheek raiser and lid compressor). These reactions would have acted as mimicking behavior and, consequently, favored the recognition of disgust and happiness, respectively.

Using this framework, why did the aversive odor context facilitate the perception of anger? It is possible that the response of multisensory and emotional brain regions for each emotion is not as discrete as proposed. For example, the amygdala was first specifically involved in the processing of fear [71], but later studies showed that it is also reactive to other expressions ([46,72,73]; see also [74]). The same has occurred for other regions and expressions, with dissociable but also largely overlapping and interlocking networks for different expression categories (e.g., [75]; see also [66,76]). Thus, the aversive-anger relation observed here indicates that the cognitive processes elicited to categorize anger and disgust share, at least partly, similar cerebral networks that are activated by the presence of an aversive odor. However, we noted that the effect of the aversive odor on the intensity of expression for correct perception tended to be larger for disgust when the emotion names were provided and larger for anger when they were not. Likewise, the aversive odor reduced intrusions for disgust but increased intrusions for anger only in the presence of verbal information. Thus, the overlap in the perception of disgust and anger may depend on the presence of verbal information, as verbal cues may act as a shaping context for more discrete emotional categories. Accordingly, the pleasant odor context elicited greater intrusions for all negative emotions only when the emotion names were not provided. When no verbal information shapes the emotion categories as discrete entities, the different expressions may be more organized along a positive-negative dimension by the pleasant odor context.

In a similar way, the motor/mirror neurons hypothesis may explain why the aversive odor context influenced both the perception of disgust and anger. Both expressions share common action units and look very similar ([57,58]; see also [77,78]). In this way, mistaken, disgusted and angry faces are among the most frequent confusions (e. g., [59]). In the framework of the odor context, the aversive odorant may have pre-activated common facial units for disgust and anger (e.g., brow lowered, upper lip raised, chin raised, lip parted, jaw dropped), lowering the perception threshold for both facial expressions. Again, this mechanism may have been modulated by the presence of the emotion names. When they were provided, a cumulative effect with the aversive odor context may have occurred, thus orienting more toward the difference in facial units for disgust and anger, and explaining the opposite effects for intrusions on the disgust and anger continua. In other words, a mimicking-like behavior elicited by the aversive odor may have been more finely shaped toward the facial configuration of disgust when participants were also confronted with the “disgust” category in verbal cues.

It must be noted that the threshold for happiness was lower than the threshold for the other expressions, despite our attempt to equate these thresholds for all expressions. Such threshold equalization worked well for negative expressions but not for the positive one, a result that can be explained as follows. First, happiness is frequently described as the easiest expression to recognize, the well-known “happy face advantage” classically observed in reaction times (e.g., [53]). Thus, the threshold was lower in our pilot study for happiness than for any other expression, as was already reported in previous studies (e.g., [79]). A possible consequence of this phenomenon is that we may have failed to adjust the threshold for this expression due to the ceiling effect for happiness in the pilot study. Nevertheless, as previously underlined, this ceiling effect was not specific to our material but reflects a property of this emotion category. Second, happiness was the single positive expression, and it was contrasted with four negative expressions. This configuration may have induced a frequency effect imbedded in a decisional bias; participants may have waited for equivalent positive and negative expressions, so a low occurrence of positive happy faces drove an overestimation of happy faces (for discussion of a potential positive bias, see [53]). This phenomenon may have increased the happy response and, consequently, reduced the threshold for happiness. Finally, and linked to both previous points, happiness is the expression that is least mistaken for others, especially in comparison with anger, disgust, and sadness which are frequently confused. Thus, any attempt to standardize the threshold for happiness and negative expressions may fail or need the use of very low levels of happiness relative to other emotions. Regardless of cause, differences in thresholds did not prevent the effects of the odor context, nor could they explain the congruency effects between the emotional meaning of the odor and of the facial expressions reported here.

In conclusion, olfaction plays a role in the visual processing of the emotional environment. More specifically, olfaction helps to clarify low-intensity expressive faces by lowering the threshold of perception for expressions that are emotionally congruent with the odor context. This influence takes place in the framework of other types of contextual information, with influences from verbal information.

## Supporting Information

S1 Fig Intensity of expression for correct perception. Mean minimum intensity of expression (in percentage of expression) in the morphed target faces for correct perception of the expression, according to Odor context, Expression continua, and Group (error bars are standard errors of the means).
(TIF)

S2 Fig Percentage of times a given expression intruded. Mean percentages of times a given expression intruded in other continua according to Odor context, Intruding expression, and Group (error bars are standard errors of the means).
(TIF)

S1 Table Intensity of expression for correct perception: individual data. (PDF)

S2 Table Percentage of intrusions: individual data. (PDF)

S3 Table Percentage of times a given expression intruded: individual data. (PDF)
