# Supplementary figures and images for: The Odor Context Facilitates the Perception of Low-Intensity Facial Expressions of Emotion
Source: PLoS One. 2015 Sep 21;10(9):e0138656. doi: 10.1371/journal.pone.0138656 (PMC4577100; doi:10.1371/journal.pone.0138656)

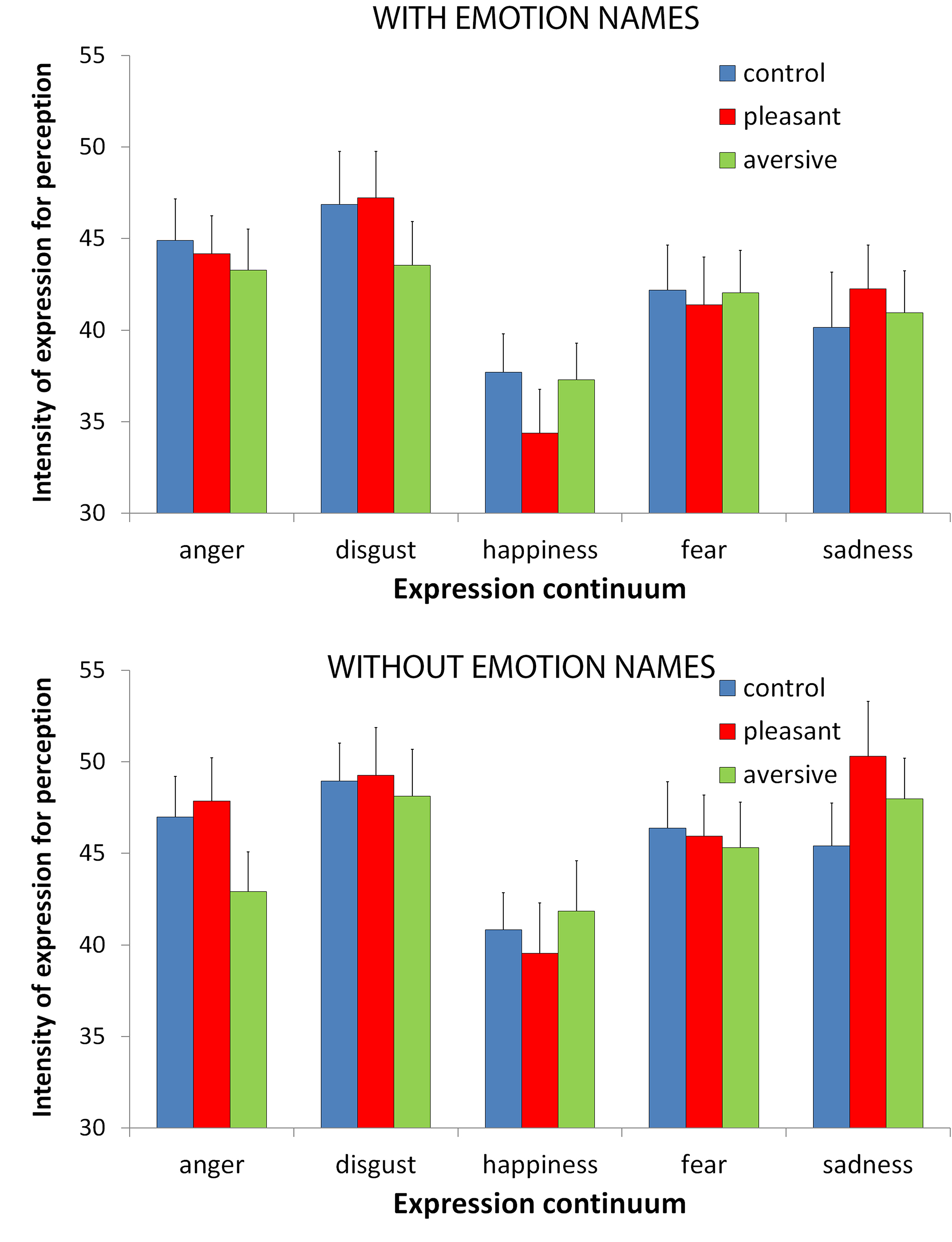

Supplement: S1 Fig — Mean minimum intensity of expression (in percentage of expression) in the morphed target faces for correct perception of the expression, according to Odor context, Expression continua, and Group (error bars are standard errors of the means). (TIF) [file pone.0138656.s001.tif]

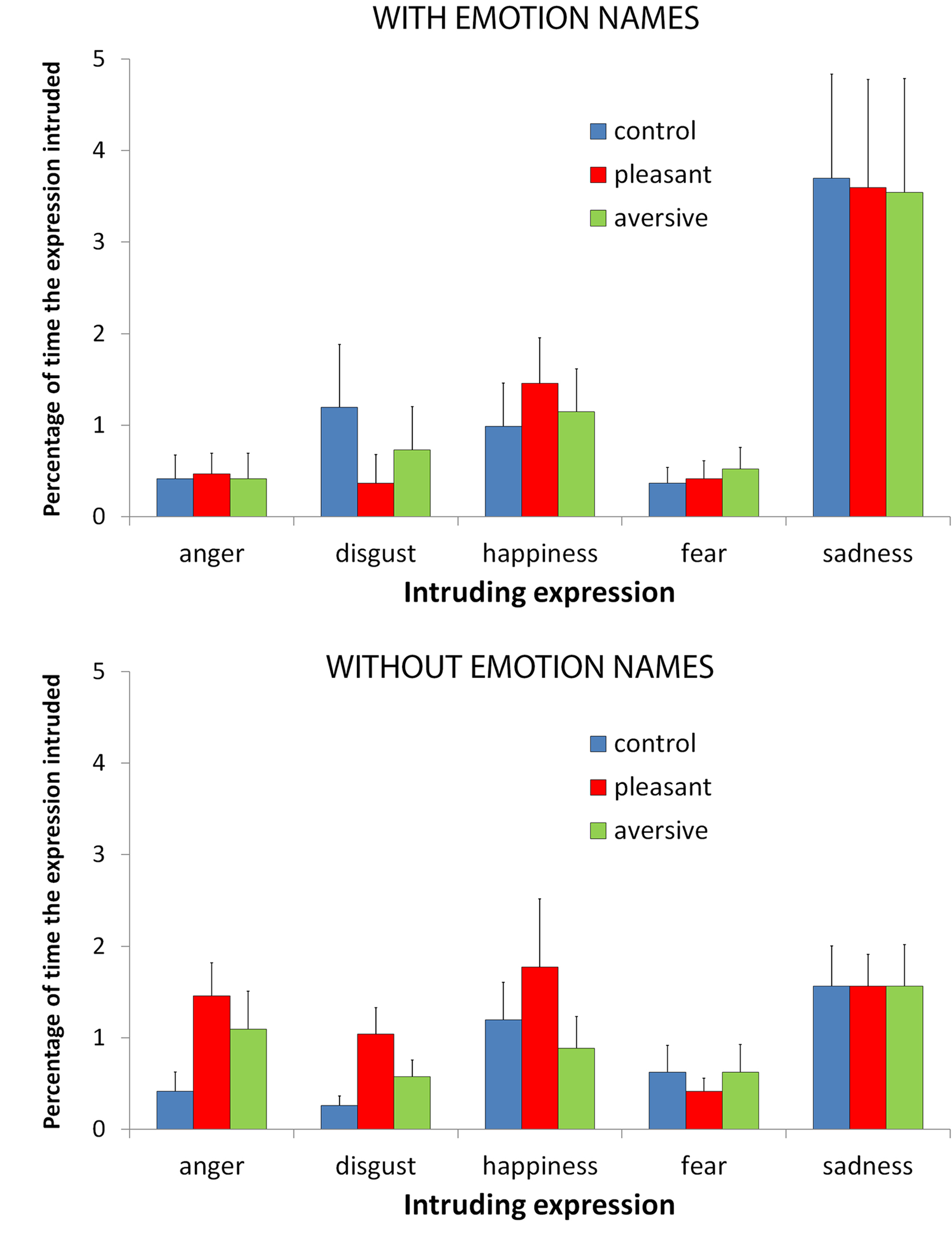

Supplement: S2 Fig — Mean percentages of times a given expression intruded in other continua according to Odor context, Intruding expression, and Group (error bars are standard errors of the means). (TIF) [file pone.0138656.s002.tif]
