# Supplementary material for: The Odor Context Facilitates the Perception of Low-Intensity Facial Expressions of Emotion
Source: PLoS One. 2015 Sep 21;10(9):e0138656. doi: 10.1371/journal.pone.0138656 (PMC4577100; doi:10.1371/journal.pone.0138656)
Supplement: S1 Table — (PDF) [file pone.0138656.s003.pdf]

Intensity of expression for correct perception

Subject	Group	no odor anger	disgust	happiness	fear	sadness	pleasant anger	disgust	happiness	fear	sadness	aversive anger	disgust	happiness	fear	sadness
1	without emotion names	45	30	30	35	25	40	35	30	30	20	45	35	50	35	42,5
2	without emotion names	50	40	50	40	40	45	25	45	45	65	45	30	50	40	50
3	without emotion names	30	50	20	50	35	20	67,5	10	40	25	20	62,5	10	37,5	30
4	without emotion names	40	30	30	37,5	30	35	25	30	35	35	35	25	30	30	35
5	without emotion names	55	55	45	40	65	60	55	50	35	50	50	60	50	40	55
6	without emotion names	50	40	30	25	47,5	65	50	25	35	50	50	45	30	35	55
7	without emotion names	60	55	45	65	65	70	57,5	40	65	77,5	65	57,5	45	70	55
8	without emotion names	45	50	30	20	40	50	50	30	37,5	80	50	40	40	25	65
9	without emotion names	67,5	40	40	55	45	50	40	35	45	40	45	30	45	50	40
10	without emotion names	45	45	50	48,33333333	40	57,5	40	40	45	40	40	45	40	35	25
11	without emotion names	40	30	45	25	45	35	35	50	35	55	30	30	35	30	45
12	without emotion names	30	45	25	35	25	40	30	25	35	57,5	30	30	15	25	35
13	without emotion names	50	50	50	50	40	45	55	45	50	45	40	55	55	60	50
14	without emotion names	50	55	35	60	50	50	60	15	55	55	55	60	29,16666667	55	50
15	without emotion names	55	55	50	45	50	50	60	65	70	40	47,5	55	60	50	45
16	without emotion names	35	50	55	50	40	40	40	50	45	45	30	45	60	60	40
17	without emotion names	60	60	55	67,5	55	60	70	48,33333333	65	55	55	55	55	60	65
18	without emotion names	35	60	50	55	45	57,5	62,5	65,83333333	55	60	40	60	60	60	50
19	without emotion names	50	50	35	55	50	60	50	42,5	50	50	45	60	45	50	66,66666667
20	without emotion names	50	50	40	45	45	46,66666667	55	40	40	52,5	35	55	35	45	45
21	without emotion names	45	50	50	50	45	46,66666667	50	40	60	45	40	50	40	50	50
22	without emotion names	50	70	35	60	50	35	60	35	40	45	45	45	30	50	50
23	without emotion names	65	55	45	50	72,5	55	55	52,5	50	75	60	65	55	50	62,5
24	without emotion names	25	60	40	50	45	35	55	40	40	45	32,5	60	40	45	45
25	with emotion names	35	15	20	40	30	35	25	10	40	30	40	25	30	40	40
26	with emotion names	65	55	57,5	60	57,5	65	50	50	55	40	60	50	50	55	55
27	with emotion names	35	55	45	37,5	25	30	50	40	30	40	30	50	45	31,66666667	30
28	with emotion names	40	55	30	45	30	35	45	30	35	45	40	50	35	35	35
29	with emotion names	40	35	30	40	22,5	25	35	20	35	10	30	35	20	35	45
30	with emotion names	60	55	40	55	55	52,5	55	47,5	62,5	57,5	50	50	35	60	65
31	with emotion names	40	40	45	30	45	40	40	50	30	50	40	40	50	47,5	45
32	with emotion names	32,5	40	35	55	56,25	45	50	40	38,33333333	51,66666667	58,33333333	22,5	35	55	52,5
33	with emotion names	40	40	55	35	30	45	40	40	35	37,5	35	35	45	40	35
34	with emotion names	55	65	30	55	62,5	45	65	15	40	40	50	60	20	40	45
35	with emotion names	45	50	30	30	30	35	45	25	25	45	40	50	30	35	30
36	with emotion names	80	85	40	55	65	65	83,33333333	50	55	55	70	70	50	60	60
37	with emotion names	55	45	40	45	25	45	45	32,5	45	35	55	35	40	35	25
38	with emotion names	45	50	50	45	35	50	45	40	62,5	40	45	45	40	45	30
39	with emotion names	50	45	35	40	50	40	50	40	40	35	40	45	35	35	40
40	with emotion names	40	60	25	40	25	35	55	25	40	30	35	50	30	35	30
41	with emotion names	40	30	17,5	10	27,5	40	40	15	10	40	35	35	25	10	45
42	with emotion names	40	35	45	45	40	55	40	40	50	45	40	35	50	45	40
43	with emotion names	45	40	40	45	15	50	40	40	50	40	40	40	35	50	20
44	with emotion names	35	40	35	35	50	40	45	35	45	40	30	40	35	40	40
45	with emotion names	35	30	30	20	35	42,5	30	25	25	35	30	25	25	30	30
46	with emotion names	45	45	40	45	40	45	45	30	40	45	35	45	40	50	45
47	with emotion names	40	55	50	45	62,5	40	45	45	45	70	50	50	55	45	55
48	with emotion names	40	60	40	60	50	60	70	40	60	57,5	60	62,5	40	55	45
