# Supplementary material for: The Odor Context Facilitates the Perception of Low-Intensity Facial Expressions of Emotion
Source: PLoS One. 2015 Sep 21;10(9):e0138656. doi: 10.1371/journal.pone.0138656 (PMC4577100; doi:10.1371/journal.pone.0138656)
Supplement: S2 Table — (PDF) [file pone.0138656.s004.pdf]

Percentage of intrusions

Subject	Group	no odor anger	disgust	happiness	fear	sadness	pleasant anger	disgust	happiness	fear	sadness	aversive anger	disgust	happiness	fear	sadness
1	without emotion names	0	0	0	0	0	0	0,05	0	0	0	0,05	0	0	0	0
2	without emotion names	0	0	0	0	0	0	0	0	0	0	0	0	0	0	0
3	without emotion names	0	0,15	0	0,05	0	0	0,2	0,05	0,15	0	0	0,3	0	0	0
4	without emotion names	0	0	0	0,05	0	0,05	0,1	0	0	0,05	0,1	0	0	0,05	0,05
5	without emotion names	0	0	0	0	0	0,05	0	0	0	0	0	0	0	0	0
6	without emotion names	0	0	0	0	0	0,05	0,05	0	0	0	0,05	0	0	0,1	0
7	without emotion names	0,05	0,05	0,05	0,25	0	0,05	0,05	0	0,2	0,2	0	0,05	0	0,1	0
8	without emotion names	0	0,1	0	0	0	0,05	0,15	0	0	0,05	0,05	0,1	0	0	0,1
9	without emotion names	0,05	0	0	0,05	0	0	0	0	0	0	0	0	0	0	0
10	without emotion names	0	0	0	0,1	0	0,15	0	0	0	0	0	0,05	0,05	0	0
11	without emotion names	0	0	0	0	0	0,05	0,05	0	0	0	0	0	0	0	0
12	without emotion names	0	0,1	0,05	0	0	0	0	0	0	0,05	0	0	0	0	0,05
13	without emotion names	0	0,05	0	0	0	0	0,1	0	0	0,05	0	0,1	0	0	0
14	without emotion names	0	0,05	0	0,15	0	0,1	0,2	0	0,25	0,15	0	0,15	0,05	0,15	0,05
15	without emotion names	0	0,25	0,05	0,05	0	0	0,25	0,15	0,2	0,05	0	0,15	0,1	0	0
16	without emotion names	0	0,2	0,15	0	0	0	0,1	0	0,05	0	0	0,05	0,2	0	0
17	without emotion names	0	0,15	0	0,05	0,05	0	0,25	0	0	0,05	0,05	0,1	0	0,05	0,1
18	without emotion names	0	0,25	0	0	0	0,1	0,1	0,1	0	0,2	0	0,1	0,05	0	0
19	without emotion names	0	0,1	0	0	0	0	0,15	0,05	0	0	0	0,2	0,05	0	0,05
20	without emotion names	0	0,05	0	0	0,05	0	0,1	0,05	0	0,1	0	0,15	0	0,05	0,1
21	without emotion names	0	0,1	0	0	0	0,05	0	0	0	0,2	0	0,05	0	0	0,05
22	without emotion names	0	0,3	0	0	0,05	0	0,2	0	0	0	0	0,1	0	0	0,05
23	without emotion names	0	0,1	0,05	0	0,1	0	0,15	0,15	0	0,2	0	0,2	0,1	0,05	0,05
24	without emotion names	0	0,25	0	0	0,2	0	0,15	0	0	0,15	0,05	0,3	0	0	0,25
25	with emotion names	0,1	0	0	0,05	0,05	0	0,05	0	0,05	0	0,1	0	0	0	0
26	with emotion names	0,05	0,05	0	0,05	0,1	0	0,05	0	0	0,05	0	0,05	0,05	0,05	0,1
27	with emotion names	0,05	0,4	0,05	0	0	0,05	0,3	0,05	0	0	0,05	0,3	0,1	0,05	0
28	with emotion names	0	0,2	0	0,05	0	0	0,1	0	0	0	0	0,15	0	0	0
29	with emotion names	0	0,05	0	0,1	0	0	0,1	0	0	0	0	0,1	0	0,1	0,05
30	with emotion names	0	0,1	0	0	0	0	0,15	0,05	0	0,05	0	0,05	0	0,05	0
31	with emotion names	0	0	0	0	0,05	0	0,05	0	0	0,05	0,05	0,05	0	0	0
32	with emotion names	0,15	0,8	0,15	0,3	0,5	0,2	0,65	0,25	0,25	0,35	0,3	0,85	0,15	0,35	0,35
33	with emotion names	0	0	0	0	0	0	0	0	0	0	0	0	0	0	0
34	with emotion names	0	0,45	0	0,05	0,05	0	0,45	0	0,05	0	0	0,4	0	0,05	0
35	with emotion names	0,1	0,2	0,05	0	0	0,05	0,15	0	0	0	0,15	0,15	0,05	0,05	0
36	with emotion names	0	0,45	0	0	0	0	0,45	0	0	0	0,05	0,35	0	0	0
37	with emotion names	0	0	0	0	0	0	0,05	0	0	0	0	0	0	0	0,05
38	with emotion names	0	0,05	0	0	0	0	0,05	0	0,05	0	0	0,05	0	0	0
39	with emotion names	0	0,15	0	0	0	0	0,15	0	0	0	0	0,1	0	0	0
40	with emotion names	0	0,3	0	0	0	0	0,3	0	0	0	0	0,15	0	0	0
41	with emotion names	0	0	0,05	0	0	0	0,05	0	0	0	0,05	0,05	0	0	0,05
42	with emotion names	0	0,1	0	0,1	0	0	0,1	0	0	0	0,05	0,05	0,05	0	0
43	with emotion names	0,15	0,15	0,1	0,05	0	0,1	0,25	0,15	0,2	0,05	0,15	0,1	0,05	0,15	0
44	with emotion names	0	0,05	0	0	0	0	0,1	0	0	0	0,05	0	0	0	0
45	with emotion names	0	0	0	0	0	0,05	0	0	0	0	0	0	0	0	0
46	with emotion names	0	0	0	0	0	0	0,05	0	0	0	0	0,05	0	0	0
47	with emotion names	0	0,15	0	0	0,1	0	0,1	0	0	0	0	0,05	0	0	0
48	with emotion names	0	0,15	0	0	0	0	0,25	0	0	0	0	0,15	0	0	0
