# Supplementary material for: The Odor Context Facilitates the Perception of Low-Intensity Facial Expressions of Emotion
Source: PLoS One. 2015 Sep 21;10(9):e0138656. doi: 10.1371/journal.pone.0138656 (PMC4577100; doi:10.1371/journal.pone.0138656)
Supplement: S3 Table — (PDF) [file pone.0138656.s005.pdf]

Percentage of times a given expression intruded

Subject	Group	no odor anger	disgust	happiness	fear	sadness	pleasant anger	disgust	happiness	fear	sadness	aversive anger	disgust	happiness	fear	sadness
1	without emotion names	0	0	0	0	0	0,0125	0	0	0	0	0	0,0125	0	0	0
2	without emotion names	0	0	0	0	0	0	0	0	0	0	0	0	0	0	0
3	without emotion names	0	0	0	0	0,05	0,05	0	0,025	0	0,025	0,0375	0	0,0125	0	0,025
4	without emotion names	0	0	0	0	0,0125	0	0,025	0	0	0,025	0	0,025	0	0,0125	0,0125
5	without emotion names	0	0	0	0	0	0	0,0125	0	0	0	0	0	0	0	0
6	without emotion names	0	0	0	0	0	0	0	0	0	0,025	0	0,025	0	0	0,0125
7	without emotion names	0	0	0,075	0,025	0	0,05	0,0125	0,05	0	0,0125	0,0125	0	0,025	0	0
8	without emotion names	0,025	0	0	0	0	0,0375	0,0125	0	0,0125	0	0,0375	0	0	0,0125	0,0125
9	without emotion names	0	0,0125	0,0125	0	0	0	0	0	0	0	0	0	0	0	0
10	without emotion names	0	0,0125	0,0125	0	0	0	0,025	0	0	0,0125	0,025	0	0	0	0
11	without emotion names	0	0	0	0	0	0	0	0,025	0	0	0	0	0	0	0
12	without emotion names	0	0	0,0125	0	0,025	0	0	0	0,0125	0	0	0	0,0125	0	0
13	without emotion names	0	0	0	0	0,0125	0,0125	0	0,025	0	0	0,0125	0	0	0,0125	0
14	without emotion names	0	0	0,0375	0	0,0125	0	0,0125	0,15	0	0,0125	0	0	0,075	0	0,025
15	without emotion names	0	0,0125	0,0375	0,025	0,0125	0,025	0,0125	0,1	0,025	0	0	0	0,0125	0,0375	0,0125
16	without emotion names	0	0	0,025	0,0625	0	0	0	0,0125	0,0125	0,0125	0	0	0	0,0625	0
17	without emotion names	0	0,0125	0,05	0	0	0,0375	0	0,0375	0	0	0,025	0,0125	0,0375	0	0
18	without emotion names	0	0	0,025	0	0,0375	0,025	0,05	0	0,0125	0,0375	0,0125	0,0125	0,0125	0	0
19	without emotion names	0	0	0	0	0,025	0	0	0	0,0125	0,0375	0	0,0125	0	0	0,0625
20	without emotion names	0,025	0	0	0	0	0,0125	0,025	0	0	0,025	0	0,025	0	0	0,05
21	without emotion names	0	0	0	0	0,025	0,025	0,025	0	0	0,0125	0	0,0125	0	0	0,0125
22	without emotion names	0	0	0	0,0125	0,075	0	0	0	0	0,05	0,0125	0	0	0	0,025
23	without emotion names	0,0125	0,0125	0	0,0125	0,025	0,025	0,0375	0	0,0125	0,05	0	0	0,0125	0,0125	0,075
24	without emotion names	0,0375	0	0	0,0125	0,0625	0,0375	0	0	0	0,0375	0,0875	0	0,0125	0	0,05
25	with emotion names	0	0,0375	0	0,0125	0	0,0125	0	0	0	0,0125	0	0,025	0	0	0
26	with emotion names	0,0375	0,0125	0,0125	0	0	0,0125	0	0,0125	0	0	0,025	0,0125	0,025	0	0
27	with emotion names	0	0	0	0	0,125	0	0	0	0	0,1	0	0	0	0	0,125
28	with emotion names	0	0	0,0125	0	0,05	0	0	0,0125	0	0,0125	0	0	0	0	0,0375
29	with emotion names	0	0,0125	0	0,0125	0,0125	0	0	0	0,0125	0,0125	0	0,0125	0,0125	0,0125	0,025
30	with emotion names	0	0	0,0125	0,0125	0	0	0	0,0375	0,025	0	0	0	0,025	0	0
31	with emotion names	0	0,0125	0	0	0	0	0,0125	0	0	0,0125	0	0	0	0	0,025
32	with emotion names	0,05	0,1625	0,0125	0,0375	0,2125	0,05	0,075	0,0375	0,0375	0,225	0,0625	0,1125	0,0125	0,05	0,2625
33	with emotion names	0	0	0	0	0	0	0	0	0	0	0	0	0	0	0
34	with emotion names	0,0125	0,0125	0,1125	0	0	0,0125	0	0,1125	0	0	0	0	0,1125	0	0
35	with emotion names	0	0	0	0	0,0875	0	0	0	0	0,05	0	0	0	0,0125	0,0875
36	with emotion names	0	0	0	0	0,1125	0	0	0	0	0,1125	0	0	0	0	0,1
37	with emotion names	0	0	0	0	0	0,0125	0	0	0	0	0	0	0,0125	0	0
38	with emotion names	0	0	0,0125	0	0	0	0	0,025	0	0	0	0	0,0125	0	0
39	with emotion names	0	0	0	0	0,0375	0,0125	0	0	0	0,025	0	0	0	0	0,025
40	with emotion names	0	0	0,0125	0	0,0625	0	0	0,025	0	0,05	0	0	0,0125	0	0,025
41	with emotion names	0	0	0	0,0125	0	0	0	0	0,0125	0	0	0	0,0125	0,025	0
42	with emotion names	0	0,0125	0,025	0	0,0125	0	0	0,025	0	0	0	0,0125	0,0125	0,0125	0
43	with emotion names	0	0	0	0	0,1125	0	0	0,0125	0,0125	0,1625	0,0125	0	0,0125	0	0,0875
44	with emotion names	0	0	0,0125	0	0	0	0	0,0125	0	0,0125	0	0	0	0,0125	0
45	with emotion names	0	0	0	0	0	0	0	0	0	0,0125	0	0	0	0	0
46	with emotion names	0	0	0	0	0	0	0	0,0125	0	0	0	0	0,0125	0	0
47	with emotion names	0	0,025	0	0	0,0375	0	0	0	0	0,025	0	0	0	0	0,0125
48	with emotion names	0	0	0,0125	0	0,025	0	0	0,025	0	0,0375	0	0	0	0	0,0375
